# ﻿Two new *Rinodina* lichens from South Korea, with an updated key to the species of *Rinodina* in the far eastern Asia

**DOI:** 10.3897/mycokeys.87.71524

**Published:** 2022-02-23

**Authors:** Beeyoung Gun Lee, Jae-Seoun Hur

**Affiliations:** 1 Baekdudaegan National Arboretum, Bonghwa, 36209, Republic of Korea Baekdudaegan National Arboretum Bonghwa Republic of Korea; 2 Korean Lichen Research Institute, Sunchon National University, Suncheon 57922, Republic of Korea Sunchon National University Suncheon Republic of Korea

**Keywords:** Biodiversity, corticolous, phylogeny, Physciaceae, taxonomy

## Abstract

*Rinodinasalicis* Lee & Hur and *Rinodinazeorina* Lee & Hur are described as new lichen-forming fungi from forested wetlands or a humid forest in South Korea. *Rinodinasalicis* is distinguishable from *Rinodinaexcrescens* Vain., the most similar species, by its olive-gray thallus with smaller areoles without having blastidia, contiguous apothecia, non-pruinose discs, paler disc color, wider ascospores in the *Pachysporaria*-type II, and the absence of secondary metabolites. *Rinodinazeorina* differs from *Rinodinahypobadia* Sheard by areolate and brownish thallus, non-pruinose apothecia, colorless and wider parathecium, narrower paraphyses with non-pigmented and unswollen tips, longer and narrower ascospores with angular to globose lumina, and the absence of pannarin. Molecular analyses employing internal transcribed spacer (ITS) sequences strongly support the two new species to be unique in the genus *Rinodina*. An updated key is provided to assist in the identification of all 63 taxa in *Rinodina* of the far eastern Asia.

## ﻿Introduction

*Rinodina*, the largest genus in the family Physciaceae, comprises about three hundred species worldwide (Sheard et al. 2017; Wijayawardene et al. 2020). Several infrageneric groups have been studied since Malme (1902) introduced the ascospore-type concept for the groups in *Rinodina* (Poelt 1965; Grube and Arup 2001). Although the classification based on different ascospore types has been coarsely accepted, the variety of ascospores does not always correspond to the infrageneric classification. As the pattern of ascospore ontogeny is considered more important than the spore type itself, it is understood that the ascospore types should be respected in developmental stages of a spore (Giralt 1994; Grube and Arup 2001; Sheard 2010; Resl et al. 2016).

The *Rinodina* has been studied in Europe (Mayrhofer and Poelt 1979; Giralt et al. 1995; Giralt 2001; Mayrhofer and Moberg 2002), North America (Sheard and Mayrhofer 2002; Sheard 2004, 2010, 2018; Sheard et al. 2011, 2012; Lendemer et al. 2012, 2019; Morse and Sheard 2020), islands of South America (Bungartz et al. 2016), Australia to New Zealand (Mayrhofer 1983, 1984b; Kaschik 2006; Elix 2011; Elix et al. 2020), Asia to Russian Far East (Mayrhofer 1984a; Galanina et al. 2011; Lendemer et al. 2012; Sheard et al. 2017; Galanina et al. 2018; Galanina and Ezhkin 2019; Zheng and Ren 2020; Galanina et al. 2021; Kumar et al. 2021), and South Africa (Matzer and Mayrhofer 1996; Mayrhofer et al. 2014). Molecular works have been accomplished over the continents (Grube and Arup 2001; Wedin et al. 2002; Nadyeina et al. 2010; Resl et al. 2016).

**Figure 1.**
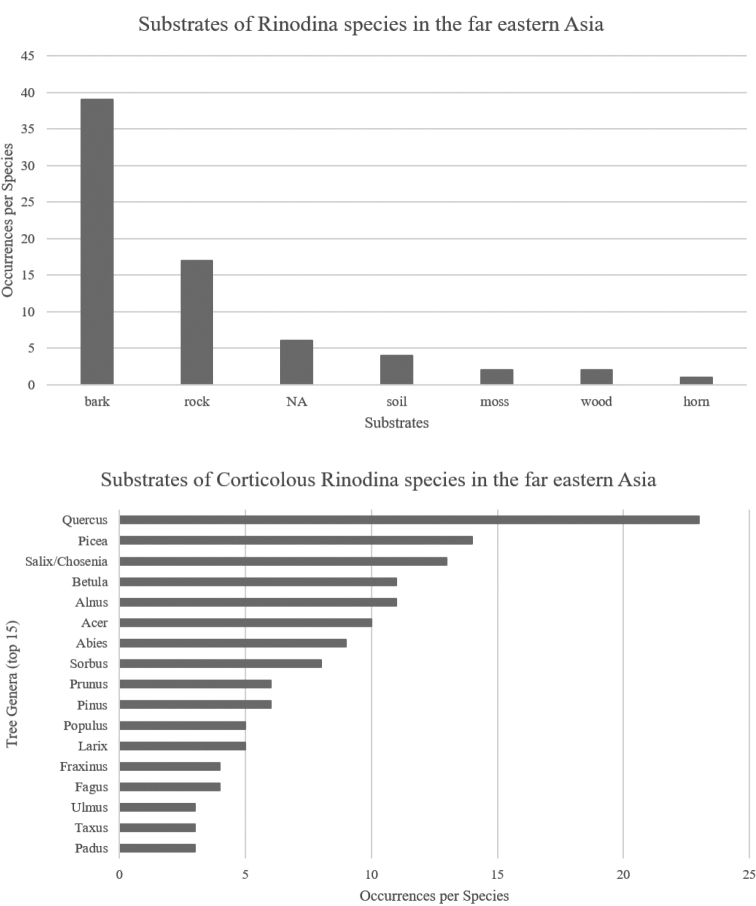
Substrates of *Rinodina* species in the far eastern Asia. *Rinodina* species of the far eastern Asia occur mainly on bark, and the genera *Quercus*, *Picea*, *Salix*, *Betula* and *Alnus* are the main substrates for corticolous *Rinodina* species of the far eastern Asia.

Sheard et al. (2017) achieved the first and comprehensive study on the genus *Rinodina* of the far eastern Asia (Korea, Japan, and Russian Far East). Several studies announced further more species in the genus, such as *R.badiexcipula* Sheard, *R.convexula* H. Magn., *R.occulta* (Körb.) Sheard, *R.oxneriana* S.Y. Kondr., Lőkös & Hur and *R.tephraspis* (Tuck.) Herre from South Korea (Kondratyuk et al. 2016, 2017; Yakovchenko 2018; Kondratyuk et al. 2020) and *R.colobinoides* (Nyl.) Müll. Arg., *R.herrei* H. Magn., *R.laevigata* (Ach.) Malme, and *R.parasitica* H. Mayrhofer & Poelt from the Kuril Islands and the Magadan region, Russian Far East (Galanina and Ezhkin 2019; Galanina et al. 2021). Among them, *R.oxneriana* was discovered as a new species and other eight species were reported as new records to the far eastern Asia. The species of *Rinodina* in the far eastern Asia are mainly corticolous and the main genera of the substrate trees are *Quercus*, *Picea*, *Salix*, *Betula* and *Alnus* (Fig. 1) (Lendemer et al. 2012; Sheard et al. 2012; Joshi et al. 2013; Kondratyuk et al. 2013, 2016, 2017, 2020; Aptroot and Moon 2014; Sheard et al. 2017; Yakovchenko et al. 2018; Galanina and Ezhkin 2019; Gananina et al. 2021). Those main substrates vigorously grow in a humid forest, a valley or a wetland, and particularly the genera *Salix* and *Alnus* often inhabit the water. Inhabiting those tree barks, diverse *Rinodina* species are easily detected in shaded forests and forested wetlands in which are one of the representative lichens of the ecosystems.

**Figure 2.**
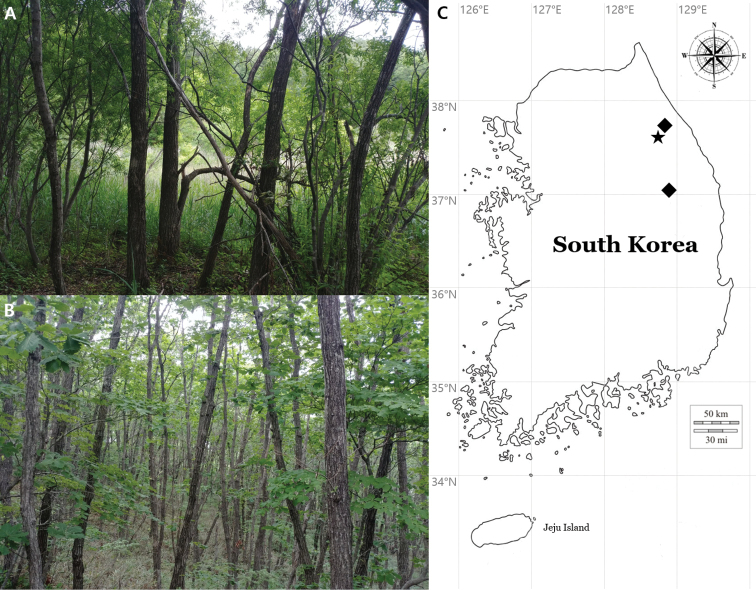
Specific collection sites for two new species **A** habitat/landscape for *R.salicis***B** habitat/landscape for *R.zeorina***C** location for *R.salicis* (a black star); locations for *R.zeorina* (two black diamonds).

This study describes two new lichen-forming fungi in the genus *Rinodina*. Field surveys for the lichen biodiversity in the forested wetlands of South Korea were carried out during the summer of 2020, and a couple of specimens of *Rinodina* were collected from barks of *Quercus* and *Salix*, the most common genera of the substrates for corticolous *Rinodina* species in the far eastern Asia, in a humid forest and a forested wetland on mountains (Fig. 2). The specimens were comprehensively analyzed in ecology, morphology, chemistry and molecular phylogeny and did not correspond to any previously known species. We describe them as new species, *Rinodinasalicis* and *R.zeorina*, and this discovery contributes to the taxonomy with overall 63 taxa in the genus *Rinodina* of the far eastern Asia. The type specimens are deposited in the herbarium of the Baekdudaegan National Arboretum (KBA, the herbarium acronym in the Index Herbariorum), South Korea.

## ﻿Materials and methods

### ﻿Morphological and chemical analyses

Hand sections were prepared manually with a razor blade under a stereomicroscope (Olympus optical SZ51; Olympus, Tokyo, Japan), scrutinized under a compound microscope (Nikon Eclipse E400; Nikon, Tokyo, Japan) and pictured using a software program (NIS-Elements D; Nikon, Tokyo, Japan) and a DS-Fi3 camera (Nikon, Tokyo, Japan) mounted on a Nikon Eclipse Ni-U microscope (Nikon, Tokyo, Japan). The ascospores were examined at 1000× magnification in water. The length and width of the ascospores were measured and the range of spore sizes was shown with average, standard deviation (SD), length-to-width ratio, and the number of measured spores. Thin-layer chromatography (TLC) was performed using solvent systems A and C according to standard methods (Orange et al. 2001).

### ﻿Isolation, DNA extraction, amplification, and sequencing

Hand-cut sections of ten to twenty ascomata per collected specimen were prepared for DNA isolation and DNA was extracted with a NucleoSpin Plant II Kit in line with the manufacturer’s instructions (Macherey-Nagel, Düren, Germany). PCR amplifications for the internal transcribed spacer region (ITS1-5.8S-ITS2 rDNA) RNA genes were achieved using Bioneer’s AccuPower PCR Premix (Bioneer, Daejeon, Korea) in 20-μl tubes with 16 μl of distilled water, 2 μl of DNA extracts and 2 μl of the primers ITS5 and ITS4 (White et al. 1990). The PCR thermal cycling parameters used were 95 °C (15 sec), followed by 35 cycles of 95 °C (45 sec), 54 °C (45 sec), and 72 °C (1 min), and a final extension at 72 °C (7 min) based on Ekman (2001). The annealing temperature was occasionally altered by ±1 degree in order to get a better result. PCR purification and DNA sequencing were accomplished by the genomic research company Macrogen (Seoul, Korea).

### ﻿Phylogenetic analyses

All ITS sequences (Table 1) were aligned and edited manually using ClustalW in Bioedit V7.2.6.1 (Hall 1999). All missing and ambiguously aligned data and parsimony-uninformative positions were removed and only parsimony-informative regions were finally analyzed in MEGA X (Stecher et al. 2020). The final alignment comprised 974 bp in which 167 variable regions were detected. The phylogenetically informative regions were 523. Phylogenetic trees with bootstrap values were obtained in RAxML GUI 2.0 beta (Edler et al. 2019) using the maximum likelihood method with a rapid bootstrap with 1000 bootstrap replications and GTR GAMMA for the substitution matrix. The posterior probabilities were obtained in BEAST 2.6.4 (Bouckaert et al. 2019) using the GTR 121343 model, as the appropriate model of nucleotide substitution produced by the bayesian model averaging methods with bModelTest (Bouckaert and Drummond 2017), empirical base frequencies, gamma for the site heterogeneity model, four categories for gamma, and a 10,000,000 Markov chain Monte Carlo chain length with a 10,000-echo state screening and 1000 log parameters. Then, a consensus tree was constructed in TreeAnnotator 2.6.4 (Bouckaert et al. 2019) with no discard of burnin, no posterior probability limit, a maximum clade credibility tree for the target tree type, and median node heights. All trees were displayed in FigTree 1.4.2 (Rambaut 2014) and edited in Microsoft Paint. The bootstrapping and posterior probability analyses were repeated three times for the result consistency and no significant differences were shown for the tree shapes and branch values. The phylogenetic trees and DNA sequence alignments are deposited in TreeBASE under the study ID 28192. Overall analyses in the materials and methods were accomplished based on Lee and Hur (2020).

**Table 1.** Species list and DNA sequence information employed for phylogenetic analysis.

No.	Species	ID (ITS)	Voucher
1	*Amandinealignicola*	JX878521	Tønsberg 36426 (BG)
2	*Amandineapunctata*	HQ650627	AFTOL-ID 1306
3	*Buelliabadia*	MG250192	TS1767 (LCU)
4	*Buelliaboseongensis*	MF399000	KoLRI 041680
5	*Buellianumerosa*	LC153799	CBM:Watanuki:L01034
6	*Rinodinaafghanica*	MT260860	500103 (XJU-L)
7	*Rinodinaalba*	GU553290	GZU 000272655
8	*Rinodinaalbana*	GU553297	GZU 000272651
9	*Rinodinaanomala*	MN587028	Sipman 62934
10	*Rinodinaarchaea*	DQ849292	H. Mayrhofer 15752 (GZU)
11	*Rinodinaatrocinerea*	AF540544	H. Mayrhofer 13.740 & U. Arup (GZU)
12	*Rinodinabalanina*	KY266842	O-L-195705
13	*Rinodinabischoffii*	DQ849291	M. Lambauer 0031 (GZU)
14	*Rinodinacacaotina*	DQ849295	H. Mayrhofer 10770 (HO)
15	*Rinodinacalcarea*	GU553292	GZU 000272654
16	*Rinodinacana*	MN587029	Sipman 63008
17	*Rinodinacapensis*	DQ849296	W. Obermayer 09230 (GZU)
18	*Rinodinaconfragosa*	DQ849297	W. Obermayer 09091 (GZU)
19	*Rinodinaconfragosula*	DQ849298	M. Lambauer 0044 (GZU)
20	*Rinodinadegeliana*	KX015681	Tønsberg 42631
21	*Rinodinadestituta*	KT695382	BIOUG24047-H02
22	*Rinodinadisjuncta*	MK812529	TRH-L-15387
23	*Rinodinaefflorescens*	KX015683	Malicek 5462
24	*Rinodinaexigua*	GU553294	GZU 000272652
25	*Rinodinagallowayi*	DQ849299	M. Lambauer 0125 (GZU)
26	*Rinodinagennarii*	AJ544187	B44435
27	*Rinodinaglauca*	GU553295	GZU 000272662
28	*Rinodinaherteliana*	DQ849300	M. Lambauer 0177 (GZU)
29	*Rinodinaimmersa*	DQ849301	M. Lambauer 0129 (GZU)
30	*Rinodinainterpolata*	AF250809	M263
31	*Rinodinajamesii*	DQ849303	H. Mayrhofer 10810 (GZU)
32	*Rinodinalecanorina*	AF540545	H. Mayrhofer 13.120 (GZU)
33	*Rinodinalepida*	AY143413	Trinkaus 137
34	*Rinodinaluridata*	DQ849304	H. Mayrhofer 12122 (GZU)
35	*Rinodinaluridescens*	AJ544183	B42835
36	*Rinodinametaboliza*	MT260864	20080224 (XJU-L)
37	*Rinodinamilvina*	GU553299	KW 63379
38	*Rinodinamniaroea*	KX015689	Spribille 20101 (GZU)
39	*Rinodinamniaroea*	KX015691	V. Wagner, 15.07.06/1 (GZU)
40	*Rinodinamniaroea*	KX015692	Spribille 20391 (GZU)
41	*Rinodinamoziana*	DQ849307	H. Mayrhofer 6729 (GZU)
42	Rinodinamozianavar.moziana	DQ849305	M. Lambauer 0214 (GZU)
43	*Rinodinanimisii*	AJ544184	B42685
44	*Rinodinaobnascens*	AJ544185	B42477
45	*Rinodinaoleae*	DQ849308	M. Lambauer 0178 (GZU)
46	*Rinodinaoleae*	GU553301	GZU 000272565
47	*Rinodinaolivaceobrunnea*	AF540547	J. Romeike 2.090300 (GOET)
48	*Rinodinaorculata*	DQ849309	H. Mayrhofer 15754 (GZU)
**49**	***Rinodinaorientalis***	**MW832807**	**BDNA-L-0000284**
**50**	***Rinodinaorientalis***	**MW832808**	**BDNA-L-0000653**
**51**	***Rinodinaorientalis***	**MW832809**	**BDNA-L-0000774**
52	*Rinodinaoxydata*	DQ849313	H. Mayrhofer 11406 (GZU)
53	*Rinodinaplana*	AF250812	E34
54	*Rinodinapyrina*	AF540549	P. Bilovitz & H. Mayrhofer 483 (GZU)
55	*Rinodinaramboldii*	DQ849315	G. Rambold 5094 (M)
56	*Rinodinareagens*	DQ849316	M. Lambauer 0218 (GZU)
57	*Rinodinaroboris*	MK811851	O-L-206765
58	*Rinodinaroscida*	DQ849317	S. Kholod plot515 (GZU)
**59**	***Rinodinasalicis***	**MW832810**	**BDNA-L-0000558**
**60**	***Rinodinasalicis***	**MW832811**	**BDNA-L-0000560**
61	*Rinodinaseptentrionalis*	GU553303	GZU 000272561
62	*Rinodinasheardii*	MK778639	J. Malicek 10238
63	*Rinodinasheardii*	MK778640	J. Vondrak 15298 (PRA)
64	*Rinodinasophodes*	AF540550	P. Bilovitz 968 (GZU)
65	*Rinodinateichophila*	GU553305	GZU 000272659
66	*Rinodinatrevisanii*	KX015684	de Bruyn s.n. 2011 (GZU)
67	*Rinodinatunicata*	AF540551	H. Mayrhofer 13.749 & R. Ertl (GZU)
68	*Rinodinaturfacea*	AF224362	Moberg 10422
69	*Rinodinavezdae*	DQ849318	H. Mayrhofer 15757 (GZU)
**70**	***Rinodinazeorina***	**MW832812**	**BDNA-L-0000642**
**71**	***Rinodinazeorina***	**MW832813**	**BDNA-L-0000646**
**72**	***Rinodinazeorina***	**MW832814**	**BDNA-L-0000650**
**73**	***Rinodinazeorina***	**MW832815**	**BDNA-L-0000651**
**74**	***Rinodinazeorina***	**MW832816**	**BDNA-L-0000668**
**75**	***Rinodinazeorina***	**MW832817**	**BDNA-L-0000933**
76	*Rinodinazwackhiana*	AF540552	H. Mayrhofer 13.848 (GZU)
77	*Rinodinellacontroversa*	AF250814	M281
78	*Rinodinelladubyanoides*	AF250815	E29
	**Overall**	**78**	

DNA sequences which were generated in this study, i.e., two new species such as *Rinodinasalicis* and *R.zeorina*, and another compared species, *R.orientalis*, are presented in bold. All others were obtained from GenBank. The species names are followed by GenBank accession numbers and voucher information. ITS, internal transcribed spacer; Voucher, voucher information.

## ﻿Results and discussion

### ﻿Phylogenetic analyses

**Figure 3.**
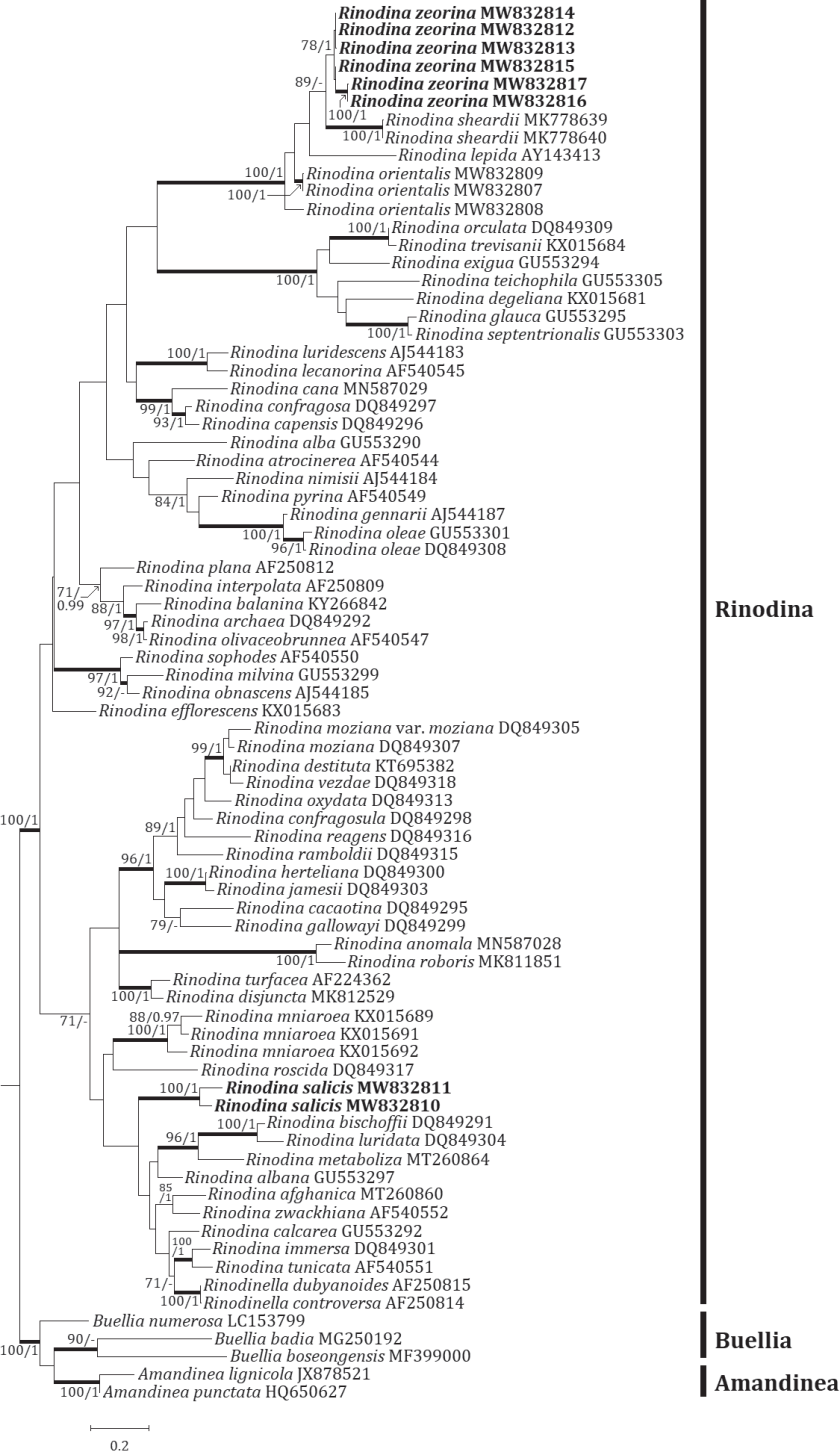
Phylogenetic relationships among available species in the genus *Rinodina* based on a maximum likelihood analysis of the dataset of ITS sequences. The tree was rooted with the sequences of the genera *Amandinea* and *Buellia*. Maximum likelihood bootstrap values ≥ 70% and posterior probabilities ≥ 95% are shown above internal branches. Branches with bootstrap values ≥ 90% are shown as fatty lines. Two new species, *R.salicis* and *R.zeorina* are presented in bold as their DNA sequences were produced from this study. All species names are followed by the Genbank accession numbers.

An independent phylogenetic tree for the genus *Rinodina* and related genera was produced from 67 sequences from GenBank and 11 newly generated sequences for the two new species and related species (Table 1). The two new species were positioned in the genus *Rinodina* in the ITS tree. The ITS tree describes that *R.salicis*, a new species, is coming alone in a single clade. Several species such as *R.mniaroea* (Ach.) Körb., *R.roscida* (Sommerf.) Arnold, *R.bischoffii* (Hepp) A. Massal., *R.luridata* (Körb.) H. Mayrhofer, Scheid. & Sheard, *R.metaboliza* Vain., *R.albana* (A. Massal.) A. Massal., *R.afghanica* M. Steiner & Poelt, *R.zwackhiana* (Kremp.) Körb., *R.calcarea* (Hepp ex Arnold) Arnold, *R.immersa* (Körb.) J. Steiner, *R.tunicata* H. Mayrhofer & Poelt, *Rinodinellacontroversa* (A. Massal.) H. Mayrhofer & Poelt, and *R.dubyanoides* (Hepp) H. Mayrhofer & Poelt, are situated close to the new species; this particular clade lacks statistical support (bootstrap value of 58 and a posterior probability of 0.75). *Rinodinazeorina*, the other new species, was located in a clade with *R.sheardii* Tønsberg, represented by a bootstrap value of 89 and a posterior probability of 0.88 (not shown) for the branch (Fig. 3).

### ﻿Taxonomy

#### ﻿ Rinodina salicis

Taxon classificationFungiCalicialesPhysciaceae

B.G. Lee & J.-S. Hur sp. nov.

1B918156-AB08-58A8-890C-E6E52C49DFCB

MB839186

Fig. 4

##### Diagnosis.

*Rinodinasalicis* differs from *R.excrescens* by olive-gray thallus with smaller areoles without blastidia, contiguous apothecia, the absence of pruina on disc, paler disc color, wider ascospores in the *Pachysporaria*-type, and the absence of secondary metabolites.

##### Type.

South Korea, Gangwon Province, Gangneung, Seongsan-myeon, Eoheul-ri, a forested wetland, 37°43.61'N, 128°48.13'E, 212 m alt., on bark of *Salixkoreensis* Andersson, 02 June 2020, B.G.Lee & H.J.Lee 2020-000358 (holotype: BDNA-L-0000558; GenBank MW832810 for ITS); same locality, on bark of *Salixkoreensis*, 02 June 2020, B.G.Lee & H.J.Lee 2020-000360, with *Caloplacagordejevii* (Tomin) Oxner, *Lecanora* sp., and *Phaeophyscia* sp. (***paratype***: BDNA-L-0000560; GenBank MW832811 for ITS).

Thallus corticolous, crustose, minutely bullate, some developing to conglomerate and continuous, rarely lobulated, thin, grayish-green to olive green, margin indeterminate, vegetative propagules absent, areoles 0.1–0.2 mm diam., 85–90 μm thick; cortex hyaline, 10 μm thick, cortical cells 5–9 μm diam.; medulla 60–65 μm thick, intermixed with algal cells, without crystals (PL–); photobiont coccoid, cells globose, 5–15 μm. Prothallus absent.

Apothecia abundant, rounded, often contiguous, emerging on the surface of thallus and sessile when mature, constricted at the base, 0.2–1.3 mm diam. Disc flat, not pruinose, pale brown or dark brown from early stages, 220–260 μm thick; margin persistent, prominent, generally entire or somewhat flexuous, a little crenulate, thalline margin concolorous to thallus but proper margin near disc distinctly pale brown. Amphithecium well-developed, with small crystals in both cortical layer and the algal-containing medulla, crystals extending to the base, not dissolving in K, 60–70 μm wide laterally, algal layers continuous to the base or solitary, algal cells 5–15 μm diam., cortical layer hyaline, 10–20 μm thick. Parathecium hyaline but light brown at periphery, 45–50 μm wide laterally and 70–80 μm wide at periphery. Epihymenium brown, not granular, pigment slightly paler in K but not diluted, 5–10 μm high. Hymenium hyaline, 70–90 μm high. Hypothecium generally hyaline, with pale yellow pigment, prosoplectenchymatous (irregular), 70–80 μm high. Oil droplets are present mainly in hypothecium and a little in hymenium. Paraphyses septate, anastomosing, 1–1.5 μm wide, simple or branched at tips, tips swollen, pigmented, epihymenium pigmented by paraphysial tips, 4.5–7.5 μm wide. Asci clavate, 8-spored, 68–90 × 20–25 μm (n = 5). Ascospores ellipsoid, 1-septate, *Pachysporaria*-type II, rarely *Physcia*-type, Type A development, hyaline when young and light brown to brown in mature, 14–24 × 8–13.5 μm (mean = 18.2 × 10.5 μm; SD = 2.12(L), 1.19(W); L/W ratio 1.2–2.4, ratio mean = 1.7, ratio SD = 0.2; n = 105). Pycnidia not detected.

**Figure 4.**
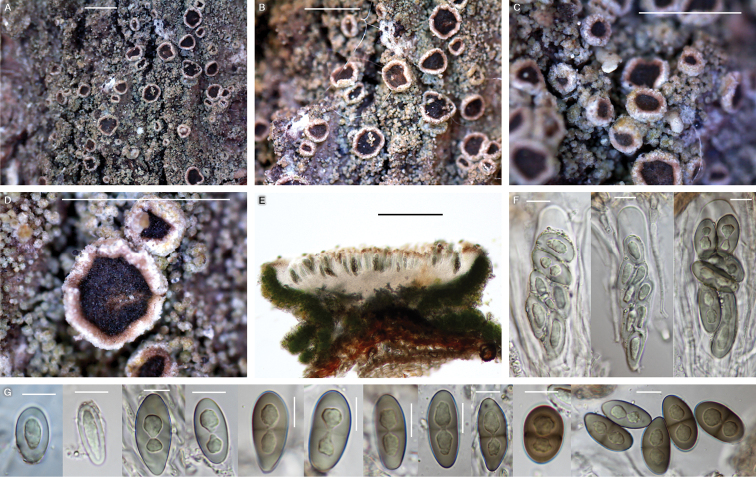
*Rinodinasalicis* (BDNA-L-0000558, holotype) in morphology **A–D** habitus and apothecia. Thallus olive-gray composed of tiny areoles and non-pruinose apothecia **E** well-developed amphithecium and algal layer extending to the base **F** asci clavate with eight spores **G** ascospores simple in the beginning and developed 1-septate, *Pachysporaria*-type II, rarely *Physcia*-type at mature. Scale bars: 1 mm (**A–D**); 200 μm (**E**); 10 μm (**F, G**).

##### Chemistry.

Thallus K–, KC–, C–, Pd–. Hymenium I+ purple-blue. UV–. No lichen substance was detected by TLC.

##### Distribution and ecology.

The species occurs on the bark of *Salixkoreensis*. The species is currently known from the type collections.

##### Etymology.

The species epithet indicates the lichen’s substrate preference, namely the substrate tree *Salixkoreensis*.

##### Notes.

The new species is similar to *R.excrescens* and *R.bullata* Sheard & Lendemer in having bullate thallus. However, the new species differs from *R.excrescens* by olive-gray thallus with smaller areoles without having blastidia, contiguous apothecia, the absence of pruina on disc, paler disc color, ascospore type, larger ascospore, and the absence of secondary metabolites (Sheard 1966; Sheard et al. 2012).

The new species is closer to *R.bullata* in having small bullate areoles without having blastidia. However, the new species differs from the latter by olive-gray thallus, contiguous and larger apothecia, proper margin with pale brown color, crystals present in both cortex and medulla in amphithecium, larger ascospores, K– reaction on thallus, and the absence of lichen substance (Sheard et al. 2012, 2017).

The new species is comparable to *R.granulans* Vain. as the latter represents thallus with minute areoles. However, the new species differs from the latter by thallus color, slightly smaller areoles without blastidia, abundance of apothecia without pruina, *Pachysporaria*-type II ascospores, K– reaction on thallus, and the absence of lichen substance (Giralt et al. 1994; Galanina et al. 2011). Reference Table 2 provides the key characteristics distinguishing *R.salicis* from the compared species above.

**Table 2.** Comparison of *Rinodinasalicis* with closely-related species.

Species	*Rinodinasalicis*	*Rinodinabullata*	*Rinodinaexcrescens*	*Rinodinagranulans*
Thallus growth form	bullate without blastidia	bullate without blastidia	bullate with blastidia	bullate with blastidia, forming leprose crust
Areoles (mm in diam.)	0.1–0.2	0.1–0.15(–0.2)	up to c. 1.98	(0.1–)0.2–0.3(–0.5)
Thallus color	olive-gray	light gray	gray	gray to gray-brown
Apothecia (mm in diam.)	0.2–1.3	0.3–0.6	up to c. 1.26	up to 0.3
Apothecia contiguation	often contiguous	not contiguous	not contiguous	not contiguous
Apothecia abundance	abundant	abundant	abundant	very rare
Pruina	absent on disc	–	often present on disc	often present on disc
Disc color	pale to dark brown	brown	brown to black	reddish brown
Proper margin	pale brown	indistinct	–	indistinct
Crystals in amphithecium	present in medulla and cortex	present in cortex	–	present
Ascospore type	*Pachysporaria*-type II	*Pachysporaria*-type II	*Physcia*-type	*Physcia*-type to *Milvina*-type
Ascospores (μm)	14–24 × 8–13.5	14.5–16.5 × 8–9	17.5–19.5 × 8.5–9.5	18–25 × 10–14
Spot test	thallus K–	thallus K+ yellow	thallus K–	thallus K+ faint yellow
Substance	absent	atranorin	pannarin, (rarely zeorin)	pannarin
Reference	BDNA-L-0000558 (holotype), BDNA-L-0000560 (paratype)	Sheard et al. 2012, 2017	Sheard 1966; Sheard et al. 2012, 2017	Giralt et al. 1994; Galanina et al. 2011

The morphological and chemical characteristics of several species close to the new species are referenced from the previous literature. All information on the new species is produced from type specimens (BDNA-L-0000558 and BDNA-L-0000560) in this study.

#### ﻿ Rinodina zeorina

Taxon classificationFungiCalicialesPhysciaceae

B.G. Lee & J.-S. Hur sp. nov.

C2162038-9969-5520-BB2F-DE0B93647AE6

MB839187

Fig. 5

##### Diagnosis.

*Rinodinazeorina* differs from *R.hypobadia* by areolate, brownish thallus, apothecia without pruina, hyaline and wider parathecium, narrower paraphyses with hyaline and unswollen tips, longer and narrower ascospores with just angular to globose lumina, and the absence of pannarin.

##### Type.

South Korea, North Gyeongsang Province, Bonghwa-gun, Seokpo-myeon, Mt. Cheongok, 37°01.89'N, 128°58.65'E, 1,104 m alt., on bark of *Quercusmongolica*, 16 June 2020, B.G. Lee & H.J. Lee 2020-000733, with *Biatora* sp., *Lecidellaeuphorea* (Flörke) Kremp., *Pertusariamultipuncta* (Turner) Nyl., and *Sagiolechia* sp. (***holotype***: BDNA-L-0000933; GenBank MW832817 for ITS).

Thallus corticolous, crustose, areolate, rimose to continuous, thin, light gray to light brownish gray, margin indeterminate or determinate with prothallus, vegetative propagules absent, 160–250 mm diam., 80–170 μm thick, areoles 0.1–0.5 mm diam.; cortex brown, 5–8 μm thick, with epinecral layer, hyaline, 3–7 μm thick; medulla 35–40 μm thick, intermixed with algal cells, without crystals (PL–); photobiont coccoid, cells globose, 5–9 μm. Prothallus absent or brownish black when present.

**Figure 5.**
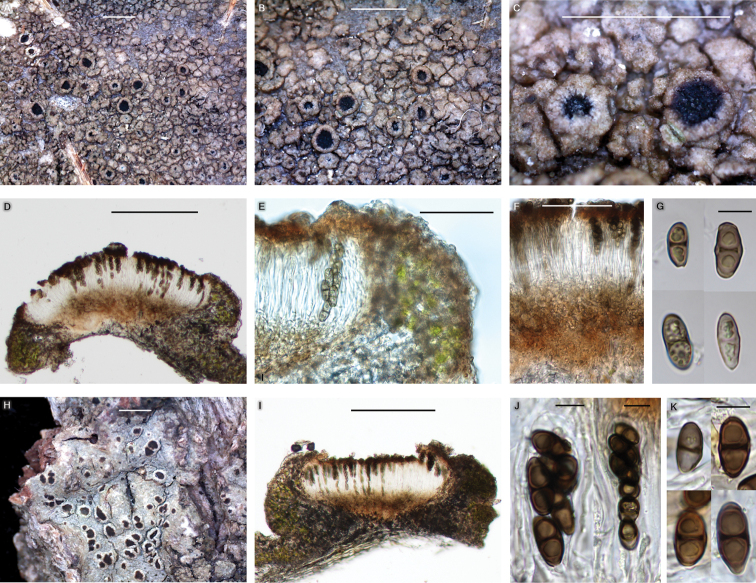
*Rinodinazeorina* (BDNA-L-0000933, holotype for **A–G**; BDNA-L-0000668 for **H–K**) in morphology **A–C** habitus and apothecia on bark of *Quercusmongolica*. Thallus brownish and areolate and non-pruinose apothecia **D** well-developed amphithecium and pigmented hypothecium **E** epihymenium with brown pigment which extending to the cortical layer of amphithecium. Parathecium light brown at periphery **F** hypothecium with light (olive-)brown pigment **G** ascospores 1-septate, *Dirinaria*-type but lumina angular to globose **H** habitus and apothecia on bark of *Tiliaamurensis*. Thallus more grayish **I** apothecial section representing well-developed amphithecium and pigmented hypothecium **J** asci clavate with eight spores **K** ascospores 1-septate, *Dirinaria*-type but lumina angular to globose. Scale bars: 1 mm (**A–C**); 200 μm (**D**); 50 μm (**E, F**); 10 μm (**G**); 1 mm (**H**); 200 μm (**I**); 10 μm (**J, K**).

Apothecia abundant, rounded, erumpent in the beginning and sessile when mature, constricted at the base, 0.2–0.6 mm diam. Disc flat, not pruinose but epinecral debris shown in water, black to dark brown from early stages, 150–200 μm thick; margin persistent, prominent, generally entire or a little crenulate, concolorous to thallus. Amphithecium well-developed, with small crystals in the algal-containing medulla and particularly near the base, dissolving in K, 70–90 μm wide laterally, algal cells evenly distributed from periphery to base, 10–15 μm diam., cortical layer brownish, cortical cells granular, 2–3 μm diam., with epinecral layer, up to 5 μm thick. Parathecium hyaline but light brown at periphery, 5–10 μm wide laterally and 20–50 μm wide at periphery. Epihymenium red-brown, small granules not dissolving in K, 8–10 μm high. Hymenium hyaline, 90–95 μm high. Hypothecium brown with olive pigment in upper part, prosoplectenchymatous (irregular), 60–65 μm high. Oil droplets present a little in hypothecium. Paraphyses septate, anastomosing, 0.5–1 μm wide, simple or branched at tips, tips generally not swollen or little swollen, not pigmented, epihymenium pigmented by small granules, not by paraphysial tips, up to 1.5 μm wide. Asci clavate, 8-spored, 60–75 × 15–21 μm (n = 3). Ascospores ellipsoid, 1-septate, *Dirinaria*-type but lumina angular to globose, Type B development not detected, septum inflated a little or not, without a torus, hyaline when young and generally brown or dark brown in mature, 11–20 × 5–8.5 μm (mean = 15.4 × 7.1 μm; SD = 1.77(L), 0.70(W); L/W ratio 1.5–3.4, ratio mean = 2.2, ratio SD = 0.3; n = 105). Pycnidia raised, asymmetric, 175–225 μm wide. Pycnoconidia bacilliform, 3–4 × 0.5 μm.

##### Chemistry.

Thallus K–, KC–, C–, Pd–. Hymenium I+ blue. UV–. Zeorin was detected by TLC.

##### Distribution and ecology.

The species occurs on the bark of *Quercusmongolica*, *Tiliaamurensis* Rupr., and *Maackiaamurensis* Rupr. & Maxim. The species is currently known from a humid forest and a forested wetland of two mountainous sites.

##### Etymology.

The species epithet indicates that the lichen’s substance, zeorin, is a major compound.

##### Notes.

The new species is similar to *R.hypobadia*, *R.sheardii*, and *R.* sp. A in having a pigmented hypothecium. However, the new species differs from *R.hypobadia* by areolate, brownish thallus, apothecia without pruina, hyaline and wider parathecium, narrower paraphyses with hyaline and unswollen tips, longer and narrower ascospores with just angular to globose lumina, and the absence of pannarin (Sheard et al. 2017).

The new species differs from *Rinodinasheardii* by the absence of vegetative propagules, and *Dirinaria*-type ascospores in smaller size (Sheard et al. 2017).

The new species differs from *Rinodina* sp. A by wider parathecium, narrower paraphyses with swollen tips, smaller ascospores *Dirinaria*-type, and the absence of pannarin (Sheard et al. 2017).

The new species can be compared with *R.manshurica* and R.aff.oleae in having erumpent apothecia, small ascospores(<21 μm long) with swollen septum among corticolous species. However, the new species differs from *R.manshurica* by crystals present in the amphithecium, wider parathecium, narrower paraphyses without swollen tips, pigmented hypothecium, and longer and narrower ascospores (Tønsberg 1992; Sheard et al. 2017).

The new species is distinguished from R.aff.oleae by narrower ascospores, and pigmented hypothecium (vs. hyaline hypothecium) (Sheard et al. 2017). Reference Table 3 provides the key characteristics distinguishing *R.zeorina* from the compared species above.

**Table 3.** Comparison of *Rinodinazeorina* with closely-related species.

**Species**	***Rinodinazeorina***	***Rinodinahypobadia***	***Rinodinamanshurica***	***Rinodinasheardii***	**Rinodinaaff.oleae**	***Rinodina* sp. A**
Thallus growth from	areolate, rimose to continuous	rimose, not areolate	rimose, rimose-areolate	±areolate to ±continuous, sorediate	continuous, rimose-areolate	continuous to areolate
Thallus color	light gray to light brownish gray	light to dark gray	gray-brown	yellow, yellow-brown, or pale brown or greenish	(dark gray to olive-green)	dark gray to gray-brown
Pruina	absent, but epinecral debris shown in water	slightly pruinose	absent	absent	(absent)	–
Parathecium color	hyaline and light brown at periphery	red-brown	–	red-brown to brown	(hyaline to brownish)	–
Parathecium at periphery (μm)	20–50	10–20	c. 20	c. 30	(up to 30)	c. 25
Paraphyses (μm)	up to 1.5	2–2.5	2.0	2.0	(1–2)	3.0
Paraphysial tips	not or little swollen, not pigmented	3–4 μm, lightly pigmented	c. 3 μm, light pigmented	c. 3 μm	–	c. 4.5 μm, pigmented
Hypothecium color	brown with olive pigment	reddish or chestnut brown	hyaline	dilute brown to red-brown	hyaline	light brown
Crystals in amphithecium	present in medulla	present in both cortex and medulla	absent	present	–	present in medulla
Ascospore type	*Dirinaria*-type with angular-globose lumina	*Dirinaria*-type with *Physcia*- or *Physconia*-like lumina	*Dirinaria*-type, with *Physcia*-like lumina	*Pachysporaria*-type I	*Dirinaria*-type with *Physcia*-like lumina	*Pachysporaria*-type I
Ascospores (μm)	11–20 × 5–8.5	12.5–18.5 × 6.5–10	14–16.5 × 7.5–8.5	16–35 × 8–17	15.5–19 × 6.5–9.5	22–28.5 × 10.5–15.5
Pycnidia	175–225	up to 300	–	–	–	–
Pycnoconidia (μm)	3–4 × 0.5	3.5 × 1.0	–	–	(4–5 × 1)	–
Substance	zeorin	pannarin, zeorin	absent	zeorin	(absent)	pannarin, zeorin
Reference	BDNA-L-0000933 (holotype), BDNA-L-0000642, BDNA-L-0000646, BDNA-L-0000650, BDNA-L-0000651, BDNA-L-0000668	Sheard et al. 2017	Sheard et al. 2017	Tønsberg 1992; Sheard et al. 2017	Joshi et al. 2013; Smith et al. 2009; Sheard et al. 2017	Sheard et al. 2017

The morphological and chemical characteristics of several species close to the new species are referenced from the previous literature. All information on the new species is produced mainly from the type specimen (BDNA-L-0000933) in comparing with additional specimens (BDNA-L-0000642, BDNA-L-0000646, BDNA-L-0000650, BDNA-L-0000651, BDNA-L-0000668) in this study . The brackets in the column of R.aff.oleae are referenced from *R.oleae* as some information of R.aff.oleae is not mentioned in the reference.

The new species is compared further with other *Rinodina* species having the substance zeorin, *R.ascociscana* (Tuck.) Tuck., *R.buckii* Sheard, *R.efflorescens* Malme, *R.luteonigra* Zahlbr., *R.subalbida* (Nyl.) Vain., *R.subminuta* H. Magn., and *R.willeyi* Sheard & Giralt. However, all of them are different from the new species because those species represent larger ascospores in *Physcia*- to *Physconia*-type for *R.ascociscana*; sorediate thallus, mostly light brown hypothecium and *Teichophila*-type ascospores and the presence of pannarin for *R.buckii*; sorediate thallus, colorless hypothecium, *Pachysporaria*-type ascospores and the presence of pannarin and secalonic acid A for *R.efflorescens*; colorless hypothecium, larger ascospores in *Pachysporaria*-type and the presence of thiomelin for *R.luteonigra*; larger spores in *Pachysporaria*-type and the presence of pannarin for *R.subalbida*; larger spores in *Physcia*-type for *R.subminuta*; sorediate thallus and the presence of pannarin for *R.willeyi* (Sheard et al. 2012, 2017).

##### Additional specimens examined.

South Korea, Gangwon Province, Pyeongchang-gun, Daegwallyeong-myeon, Heonggye-ri, a forested wetland, 37°46.00'N, 128°42.33'E, 1,047 m alt., on bark of *Maackiaamurensis*, 03 June 2020, B.G. Lee & H.J.Lee 2020-000442, with *Buelliadisciformis* (Fr.) Mudd, *Buellia* sp., *Catillarianigroclavata* (Nyl.) J. Steiner, *Lecanoramegalocheila* (Hue) H. Miyaw., *Lecanorasymmicta* (Ach.) Ach., *Lecidellaeuphorea*, and Lambiellacf.caeca (J. Lowe) Resl & T. Sprib. (BDNA-L-0000642; GenBank MW832812 for ITS); same locality, 37°46'0.02"N, 128°42'19.58"E, 1,047 m alt., on bark of *Maackiaamurensis*, 03 June 2020, B.G. Lee & H.J.Lee 2020-000446 (BDNA-L-0000646; GenBank MW832813 for ITS); same locality, 37°46.00'N, 128°42.33'E, 1,047 m alt., on bark of *Maackiaamurensis*, 03 June 2020, B.G. Lee & H.J.Lee 2020-000450 (BDNA-L-0000650; GenBank MW832814 for ITS); same locality, 37°46.00'N, 128°42.33'E, 1,047 m alt., on bark of *Maackiaamurensis*, 03 June 2020, B.G. Lee & H.J.Lee 2020-000451 (BDNA-L-0000651; GenBank MW832815 for ITS); same locality, 37°46.00'N, 128°42.33'E, 1,047 m alt., on bark of *Tiliaamurensis*, 03 June 2020, B.G. Lee & H.J.Lee 2020-000468, with *Amandineapunctata* (Hoffm.) Coppins & Scheid., Bacidiaaff.beckhausii Körb., *Catillaria* sp., *Micareaprasina* Fr., *Phaeophyscialimbata* (Poelt) Kashiw., Rinodinacf.oleae Bagl., Traponoraaff.varians (Ach.) J. Kalb & Kalb (BDNA-L-0000668; GenBank MW832816 for ITS).

### ﻿Key to the species of *Rinodina* from the far eastern Asia (63 taxa)

Eleven more species have been recorded since Sheard et al. (2017), such as *Rinodinabadiexcipula*, *R.colobinoides*, *R.convexula*, *R.herrei*, *R.laevigata*, *R.occulta*, *R.oxneriana*, *R.parasitica*, *R.tephraspis*, and two new species from this study (Kondratyuk et al. 2016, 2017; Yakovchenko et al. 2018; Galanina and Ezhkin 2019; Kondratyuk et al. 2020; Galanina et al. 2021). Particularly, *R.laevigata* of Aptroot and Moon (2014) was rejected by Sheard et al. (2017), but Galanina et al. (2021) confirmed the species in the far eastern Asia. This key includes all above species except for *R.convexula* because the species was just announced for a new record to Korea without any specific description for reference (Kondratyuk et al. 2020). *Rinodinaconfragosa* (Ach.) Körb., *R.milvina* (Wahlenb.) Th. Fr., and *R.olivaceobrunnea* C.W. Dodge & G.E. Baker were reported from Korea and Russian Far East (Kondratyuk et al. 2016; Galanina et al. 2021) as expected to occur (Sheard et al. 2017). All expected species are remained with an asterisk mark(*).

Overall, 63 taxa of *Rinodina* are currently recorded or expected to the far eastern Asia (Korea, Japan and Russian Far East).

**Table:** 

1	Substratum rock	**2**
–	Substratum bark, wood, soil, decaying ground vegetation, bone or other lichens	**17**
2	Thalli with vegetative propagules	**3**
–	Thalli lacking vegetative propagules	**4**
3	Thallus effigurate, typically with isidia; when fertile spores belong to the *Physconia*-type; associated with seabird colonies; northern	***R.balanina***
–	Thallus not effigurate, vegetative propagules blastidia with budding soredia; spores *Pachysporaria*-type II; not coastal; southern	***R.placynthielloides***
4	Always maritime, typically on coastal rocks; spores *Dirinaria*-type	***R.gennarii***
–	Generally inland or occasionally maritime; spores belonging to a different type	**5**
5	Medulla orange, K+ red-violet; spores *Pachysporaria*-type I, ultimately developing satellite apical lumina	***R.cervina***
–	Medulla not orange, not K+ red-violet; spores of various types but never developing apical lumina	**6**
6	Thallus and apothecium margins K+ yellow, atranorin in cortex	**7**
–	Thallus and apothecium margins K−, atranorin absent	**10**
7	Spores with angular lumina, walls thickened at septum and apices, *Physcia*-type; proper exciple hyaline throughout, or if lightly pigmented not aeruginose (N−); thalline margin never pigmented	**8**
–	Spores with ‘hourglass’-shaped lumina, *Mischoblastia*-type; proper exciple typically aeruginose at periphery (N+ red under microscope); thalline margin often becoming pigmented	**9**
8	Apothecia 0.1–0.3 mm diam., hymenium 80–100 μm high, hypothecium 65–135 μm high, asci 75–80 × 16–19 μm, spores 17–27 × 8–13 μm	***R.confragosa***
–	Apothecia 0.6–1.5 mm diam., hymenium 55–85 μm high, hypothecium 10–55 μm high, asci 45–50 × 13–20 μm, spores 11–16 × 5–9 μm	***R.occulta***
9	Thallus plane; spores averaging <21 μm in length, rarely swollen at septum	***R.oxydata***
–	Thallus verrucose; spores averaging >21 μm in length, often swollen at septum when mature	***R.moziana*** (syn. *R.destituta*)
10	Spores elongately ellipsoid, l/w ratio c. 2.0, *Pachysporaria*-type	***R.cinereovirescens***
–	Spores broadly ellipsoid, l/w ratio <2.0, belonging to various types	**11**
11	Spores >20 μm long at maximum, *Teichophila*-type, often swollen at septum, more so in KOH	**12**
–	Spores <20 μm long, never swollen at septum, belonging to another type	**13**
12	Spores 18.5–25 × 10–12.5 μm	***R.tephraspis***
–	Spores 20–32 × 11–19 μm	***R.teichophila***
13	Spores with broad pigmented band around septum, *Bischoffii*-type	***R.bischoffii*** *
–	Spores lacking a broad pigmented band around septum, belonging to another type	**14**
14	Spores with *Physcia*-like lumina when immature, becoming rounded especially at the apices, lateral walls thin	**15**
–	Spores with rounded lumina from beginning, lateral walls relatively thick	**16**
15	Thallus thick, dark brown; spores constricted at septum when mature, *Milvina*-type; secondary metabolites absent	***R.milvina***
–	Thallus thin, gray to light brown; spores *Physconia*-type; thalline margin C+ red (under microscope), gyrophoric acid in medulla	***R.sicula***
16	Apothecial discs pruinose; spores *Pachysporaria*-type	***R.compensata***
–	Apothecial discs not pruinose; spores *Pachysporaria*- to *Milvina*-like	***R.kozukensis***
17	On soil, decaying ground vegetation, wood, bone or lichenicolous	**18**
–	Strictly corticolous or lignicolous	**27**
18	Spores 1-septate	**19**
–	Spores 3-septate or submuriform	**20**
19	Spores *Teichophila*-type	***R.herrei***
–	Spores *Physcia*-type, rarely with apical satellite lumina	**21**
20	Spores strictly 3-septate, type B development (apical wall thickened prior to septum formation); secondary metabolites absent	***R.conradii***
–	Spores 3-septate at first, typically becoming submuriform, type A development (apical wall thickening after septum formation); deoxylichesterinic acid present	***R.intermedia***
21	Strictly lichenicolous, on *Aspicilia* or *Rhizocarpon*	***R.parasitica***
–	Generally not lichenicolous	**22**
22	Sphaerophorin crystals in medulla (sometimes lichenicolous)	***R.turfacea***
–	Sphaerophorin lacking in medulla (never lichenicolous)	**23**
23	Cortex K+ yellow or medulla orange, K+ red	**24**
–	Cortex reaction absent	**25**
24	Thallus light gray; K+ yellow, atranorin in cortex	***R.mniaroeiza*** *
–	Thallus a shade of brown; medulla orange, K+ red, skyrin or other anthraquinones present	***R.cinnamomea*** *
25	Spores averaging <23 μm in length	***R.olivaceobrunnea***
–	Spores averaging >23 μm in length	**26**
26	Thallus and apothecia not pruinose; apothecial discs becoming convex, thalline margin then excluded; spores averaging 24.5–25.5 μm in length, l/w ratio 2.0–2.2	***R.mniaroea***
–	Thallus and apothecia typically pruinose; apothecial discs plane or concave, not convex, thalline margin never excluded; spores averaging 30–32 μm in length, l/w ratio 2.2–2.5	***R.roscida***
27	Vegetative propagules present	**28**
–	Vegetative propagules absent	**37**
28	Thallus typically golden yellow	**29**
–	Thallus a shade of gray or brown	**30**
29	Thallus with small, dense isidia; very rarely with apothecia; spores *Pachysporaria*-type I	***R.chrysidiata***
–	Thallus with marginal, labriform soralia, sometimes becoming pustulate; frequently, but not always, with apothecia; spores *Physcia*-type	***R.xanthophaea***
30	Phyllidia present	***R.oxneriana***
–	Blastidia or soredia present	**31**
31	Thallus mainly blastidiate, blastidia 35–60 μm diam.	***R.colobinoides***
–	Thallus generally not blastidiate, but sorediate or sometimes blastidiate	**32**
32	Blastidia present at margin, no substance, spores *Teichophila*-type	***R.herrei***
–	Soredia and/or blastidia present, atranorin or pannarin present, spores in various types	**33**
33	Thallus light gray; soralia labriform at first, soredia whitish; K+, P+ yellow, cortical atranorin present, pannarin absent	***R.subparieta*** (syn. *R.degeliana*)
–	Thallus darker gray; soredia never whitish; K−, P+ cinnabar, atranorin absent, pannarin present	**34**
34	Thallus usually of convex to bullate areoles; blastidia often present, sometimes breaking into soredia; zeorin typically absent, when fertile pannarin also in epihymenium	***R.excrescens***
–	Thallus never consisting of bullate areoles; soredia always present; zeorin typically present, pannarin never in epihymenium	**35**
35	Soredia typically yellowish, secalonic acid A present; spores *Physcia*-type when fertile, averaging <20 μm in length	***R.efflorescens***
–	Soredia never yellowish, secalonic acid A absent; spores not *Physcia*-type, averaging >20 μm in length	**36**
36	Thallus minutely verrucose, verrucae central on areoles, quickly forming raised soralia, later spreading over thallus surface; soredia >40 μm diam.; spores *Teichophila*-type	***R.buckii***
–	Thallus with plane areoles, soredia developing marginally on areoles, never raised centrally on verrucae, later spreading over thallus surface; soredia <40 μm diam.; spores *Pachysporaria*-type I	***R.willeyi***
37	Ascospores 3-septate or submuriform	**38**
–	Ascospores 1-septate, rarely with satellite apical cells	**39**
38	Spores strictly 3-septate, type B development (apical wall thickened prior to septum formation); secondary metabolites absent	***R.conradii***
–	Spores 3-septate at first, becoming submuriform, type A development (apical wall thickening after septum formation); deoxylichesterinic acid present	***R.intermedia***
39	Thallus brightly pigmented; xanthone present, UV+ orange	**40**
–	Thallus a shade of gray or brown; xanthone absent, UV−	**41**
40	Thallus citrine, thiomelin present; spores averaging 31.0–34.5×16.0–17.5 μm, *Pachysporaria*-type I; not sorediate; subtropical, Tsushima Island, Japan	***R.luteonigra***
–	Thallus golden yellow, secalonic acid A present; spores averaging 23.5–28.5×2.0–15.0 μm, *Physcia*-type; frequently sorediate; temperate, widely distributed	***R.xanthophaea***
41	Thallus K+ yellow or P+ cinnabar, atranorin or pannarin present	**42**
–	Thallus K−, P−, both atranorin and pannarin absent	**49**
42	Thallus K+ yellow, atranorin present, pannarin absent	**43**
–	Thallus P+ cinnabar, pannarin present, atranorin absent	**45**
43	Spores averaging >33 μm long, *Pachysporaria*-type I	***R.megistospora***
–	Spores averaging <33 μm long, *Physcia*- or *Physconia*-type	**44**
44	Spores averaging >26 μm long, strictly *Physcia*-type; never sorediate; distribution limited to coastal foreshores	***R.macrospora***
–	Spores averaging <26 μm long, *Physcia*- to *Physconia*-type; most frequently sorediate; distribution inland	***R.subparieta*** (syn. *R.degeliana*)
45	Hypothecium pigmented dark reddish brown; spores *Dirinaria*-type, (12–)14–16.5(–18)× (6.5–)7.0–8.5(–9.5) μm, lightly pigmented	***R.hypobadia***
–	Hypothecium never strongly pigmented; spore type otherwise	**46**
46	Spores averaging <20 μm in length, *Physcia*-type; thallus becoming bullate, often with minute blastidia	***R.excrescens***
–	Spores averaging >20 μm in length, not *Physcia*-type; thallus sometimes verrucate but never bullate or blastidiate	**47**
47	Thallus persistently plane; epihymenium lacking crystals, P−; spores averaging >29 μm	***R.tenuis*** (syn. *R.adirondackii*)
–	Thallus becoming verrucate; epihymenium with or without crystals, P+ or P−; spores averaging <29 μm	**48**
48	Epihymenium typically possessing pannarin crystals, P+ cinnabar; spores lacking apical canals; widely distributed in Japan and adjacent mainland	***R.subalbida***
–	Epihymenium lacking pannarin crystals, P−; spores with very obvious apical canals; Cheju Island, Korea	***Rinodina* sp. A**
49	Spores 16 per ascus	***R.polyspora***
–	Spores 4–8 per ascus	**50**
50	Medulla with sphaerophorin crystals, PL+	**51**
–	Medulla lacking sphaerophorin crystals, PL−	**52**
51	Thallus dark gray, typically dark brown; areoles becoming contiguous, plane, 0.40–0.55 mm wide; spores averaging 26.5–27.5 × 13.5–14.5 μm	***R.badiexcipula***
–	Thallus light gray, sometimes brownish; areoles remaining discrete, convex, 0.20–0.30 mm wide; spores averaging 23.0–25.5 × 11.5–13.5 μm	***R.cinereovirens***
52	Spores swollen at septum, more so in KOH, type B development (apical wall thickening prior to septum formation), *Dirinaria*-type	**53**
–	Spores not swollen at septum, even in KOH, type A development (apical wall thickening after septum formation), various types	**59**
53	Spores averaging >21 μm long	***R.endospora***
–	Spores averaging <21 μm long	**54**
54	Spores lacking wall thickening at maturity (septal and apical thickenings may be present briefly in immature spores)	**55**
–	Spore lumina *Physcia*-like, with persistent apical wall thickening	**56**
55	Thallus gray to ochraceous, rugose, areoles to 0.7 mm wide; apothecia to 0.8 mm in diam., discs plane, never convex; spores averaging 15.5–18.0 × 8.0–8.5 μm, l/w ratio 1.9–2.1	***R.mongolica***
–	Thallus gray, never ochraceous, continuous to rimose; apothecia to 0.30–0.50 mm in diam., discs often becoming convex; spores averaging 12.5–13.5 × 5.5–6.0 μm, l/w ratio 2.1–2.4	***R.pyrina*** *
56	Apothecia not erumpent; spores averaging 17.5–21.5 × 9–11 μm	***R.metaboliza***
–	Apothecia erumpent; spores smaller	**57**
57	Hypothecium pigmented with brown, spores 11–20 × 5–8.5 μm, zeorin present	***R.zeorina***
–	Hypothecium colorless, spores 15.5–18 × 8–9 μm, no substance	**58**
58	Spores averaging 15.5–16.0 μm in length	***R.manshurica***
–	Spores averaging 16.5–18.0 μm in length	**R.aff.oleae**
59	Spores averaging >22 μm in length	**60**
–	Spores averaging <22 μm in length	**61**
60	Margins of apothecia often radially cracked; spores *Physcia*- to *Physconia*-type	***R.ascociscana*** (syn. *R.akagiensis*, *R.melancholica*)
–	Margins of apothecia not radially cracked; spores *Pachysporaria*-type I	***R.dolichospora***
61	Spores *Pachysporaria*-type II	***R.salicis***
–	Spores *Physcia*- or *Physconia*-type	**62**
62	Spores *Physcia*- to *Physconia*-type, some lumina becoming rounded at apices, at maturity thin-walled	**63**
–	Spores strictly *Physcia*-type, apical walls remaining thick	**67**
63	Thallus dark brown, spores darkly pigmented at maturity, torus prominent; oro-arctic to coastal	**64**
–	Thallus a shade of gray, sometimes brownish, spores typically pigmented at maturity, torus present but not prominent; boreal	**66**
64	Thallus inconspicuous; apothecia mostly crowded, typically broadly attached	***R.olivaceobrunnea*** *
–	Thallus of dispersed or contiguous areoles; apothecia mostly dispersed, narrowly or broadly attached	**65**
65	Ascospores 20–21.5 × 10–11.5 μm, thallus well-developed, flat, scurfy or thick rugose areolate, apothecia broadly attached in the beginning then becoming narrow and even stipitate, discs convex when mature	***R.sibirica***
–	Ascospores 18.5–19.5 × 8.5–9.0 μm, thallus poorly developed, evanescent, thin or scabrid, sometimes areolate, apothecia broadly attached to thallus, discs typically flat	***R.laevigata***
66	Thallus thick, rugose, areolate; apothecia crowded, discs persistently plane, thalline margins persistent	***R.archaea*** *
–	Thallus thin, plane, continuous or rimose-areolate; apothecia dispersed, discs becoming convex, often excluding thalline margin	***R.trevisanii*** *
67	Spores averaging >18 μm long, zeorin present	***R.subminuta***
–	Spores averaging <18 μm long, zeorin absent	**68**
68	Apothecia erumpent at first, discs often becoming strongly convex; spores with lightly pigmented tori at maturity	***R.orientalis***
–	Apothecia never erumpent, discs persistently plane; spores with very dark, prominent tori at maturity	**69**
69	Apothecia crowded, broadly attached; thalli associated with leaf scars or other mesic microhabitats; areoles plane, contiguous, to >0.2 mm in diam.	***R.freyi***
–	Apothecia mostly scattered, narrowly attached; thalli typically in more xeric microhabitats; areoles convex, scattered, to 0.2 mm in diam.	***R.septentrionalis***

## Supplementary Material

XML Treatment for Rinodina salicis

XML Treatment for Rinodina zeorina
